# Knockdown of *CREB3* activates endoplasmic reticulum stress and induces apoptosis in glioblastoma

**DOI:** 10.18632/aging.102310

**Published:** 2019-10-13

**Authors:** Yaxin Hu, Liangzhao Chu, Jian Liu, Lei Yu, Shi-bin Song, Hua Yang, Feng Han

**Affiliations:** 1 Department of Prenatal Diagnosis, The Affiliated Hospital of Guizhou Medical University, Guiyang, Guizhou 550004, China; 2 Department of Cerebral Surgery, The Affiliated Hospital of Guizhou Medical University, Guiyang, Guizhou 550004, China; 3 Department of Gynecology and Obstetrics, Guiyang Maternal and Child Health Hospital, Guiyang, Guizhou 550003, China

**Keywords:** glioblastoma, cAMP responsive element binding protein 3, endoplasmic reticulum stress, apoptosis

## Abstract

Glioblastoma is a highly malignant type of central nervous system tumor. In the present study, the results of RNA sequencing indicated that cAMP responsive element binding protein 3 (CREB3) was upregulated in tumor tissues from patients with GBM. The cAMP responsive element binding protein 3 (CREB3) pathway is a major contributor to the malignant progression of glioblastoma. In this study, we explored the mechanisms by which CREB3 regulates the proliferation, invasion and apoptosis of glioblastoma. Pairs of glioblastoma and normal tissues were subjected to RNA sequencing. Then, qRT-PCR and Western blotting were used to detect *CREB3* levels in glioblastoma tissues and cell lines, respectively. *CREB3* was upregulated in glioblastoma tissues and cell lines. Overexpression of CREB3 promoted the proliferation and invasion of SHG-44 cells, while downregulation of *CREB3* inhibited the invasion of U251MG cells. Knockdown of *CREB3* also induced apoptosis in U251MG cells and increased the protein levels of BAX, active caspase 3, p-PERK, p-eIF2α and ATF4. An *in vivo* study in nude mice bearing U251MG cell xenografts confirmed these results. Our findings indicate that CREB3 functions as a tumor promoter in glioblastoma, and thus could serve as a treatment target in glioblastoma patients.

## INTRODUCTION

Glioblastoma is a highly complicated and malignant type of central nervous system tumor, accounting for >70% of all brain tumors [1, 2]. Glioblastoma cases can be categorized into grades I to IV, based on their histological characteristics and the World Health Organization criteria [3]. Grade IV glioblastoma, also known as glioblastoma multiforme, is characterized by strong invasiveness, microvascular proliferation and tissue ischemic necrosis [4]. Although advanced methods of surgery, chemotherapy and radiotherapy are available for glioblastoma treatment, the median survival of patients is only 12 to 15 months [5]. Furthermore, drug resistance and recurrence are still a challenge in patients with glioblastoma [6]. Therefore, novel effective therapies for glioblastoma are imminently needed.

The endoplasmic reticulum (ER) is the site of protein synthesis and folding. In response to certain stimuli, unfolded or misfolded proteins may accumulate in the ER, triggering endoplasmic reticulum stress (ERS) and the unfolded protein response (UPR) [7]. ERS is always present in cells undergoing rapid proliferation [8]. The UPR can be divided into three branches, which involve PKR-like ER kinase (PERK), inositol-requiring kinase 1 (IRE1) and activating transcription factor 6 (ATF6), respectively [9, 10]. The UPR alleviates stress and restores ER function by inhibiting translation and increasing the degradation of misfolded proteins [11]. Recent studies have indicated that ERS helps to maintain the homeostasis of glioblastoma cells, and thus is an important contributor to glioblastoma genesis and tumor progression [12–14].

The cAMP responsive element binding protein 3 (CREB3) is an ERS-related protein that is considered to be a transcriptional coregulator [15]. The CREB3 subfamily includes five basic leucine zipper transcription factors: CREB3, CREB3L1, CREB3L2, CREB3L3 and CREB3L4 [16]. All the CREB3 subfamily members appear to be involved in the UPR [17]. The CREB3 pathway has been reported to promote the malignant progression of glioblastoma [2]; however, the mechanism remains elusive.

In the current study, we performed gene ontology (GO) and Kyoto Encyclopedia of Genes and Genomes (KEGG) analyses to determine the differentially expressed genes (DEGs) in glioblastoma and adjacent normal tissues. We also investigated the effects of overexpressing or knocking down *CREB3* in glioblastoma cells to explore a new therapeutic strategy for this disease.

## RESULTS

### DEG screening in glioblastoma and normal tissues

**Figure 1:**
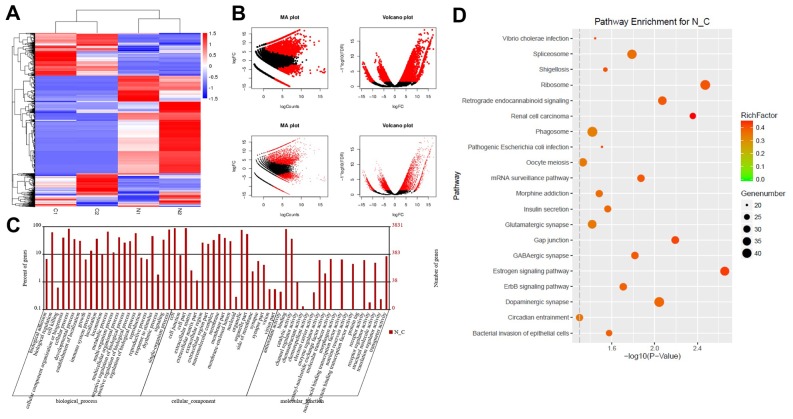
**DEG screening in glioblastoma and normal tissues.** (**A**) Heat map showing a distinguishable expression profile of genes between tumor tissues and adjacent tissues. Black represents no change in gene expression, while red represents upregulation and green represents downregulation. (**B**) The DEGs of statistical significance are represented as red points on the MA plot (log total counts versus log2 fold-change) and the volcano plot (log2 fold-change versus log false discovery rate). (**C**) DEGs were evaluated by gene ontology (GO) analysis. (**D**) DEGs were evaluated by Kyoto Encyclopedia of Genes and Genomes (KEGG) analysis.

RNA sequencing analysis was used to identify DEGs between glioblastoma and adjacent normal tissues. The downregulated and upregulated mRNAs in glioblastoma tissues compared with normal tissues are shown in Figure 1A. In addition, MA and volcano plots were generated to demonstrate the DEGs between glioblastoma and adjacent tissues, based on the thresholds of an adjusted P-value < 0.05 and a log2 fold-change ≥ 2 (Figure 1B). GO and KEGG pathway enrichment analyses were also performed, which revealed that the DEGs were mainly involved in molecular transduction and translation regulation (Figure 1C and 1D).

### The expression of DEGs in glioblastoma and normal tissues

**Figure 2:**
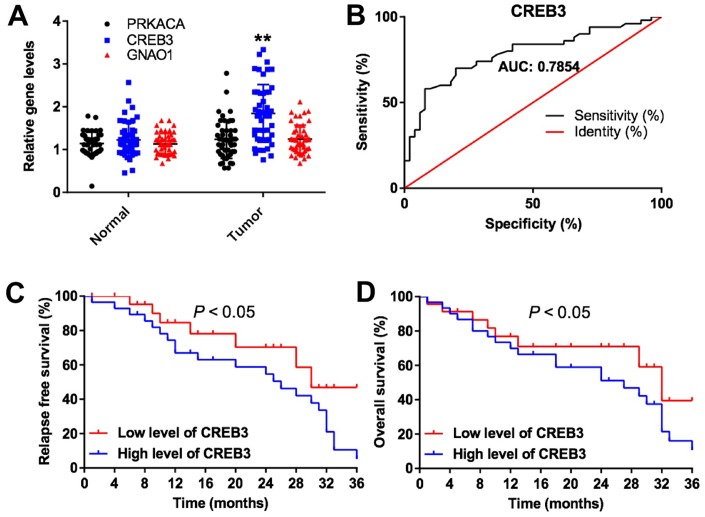
**The expression of DEGs *in vitro* and *in vivo*.** (**A**) The relative expression of *PRKACA*, *CREB3* and *GNAO1* in the tumor tissues and adjacent normal tissues of patients with glioblastoma (n = 60). (**B**) ROC curves of varying sensitivity and specificity. The closer the curve is to point “a” (x = 0, y = 100%), the more sensitive and specific the experiment is. (**C**) Pretreatment parameters as predictors of relapse-free survival in patients with glioblastoma. (**D**) The probability of overall survival in low-*CREB3* and high-*CREB3* groups. **P<0.01 compared with the normal group.

Among the DEGs, *CREB3* was obviously upregulated in glioblastoma tissues compared with adjacent normal tissues (Figure 2A). Receiver operating characteristic (ROC) curve analysis was used to discriminate glioblastoma tissue from adjacent normal tissue. The area under the curve for *CREB3* was 0.7845 in discriminating between glioblastoma and adjacent normal tissues, indicating that the experimental results were reliable (Figure 2B). As shown in Table 1, *CREB3* expression correlated with clinicopathological parameters such as the tumor volume, KI67 expression, distant metastasis and World Health Organization stage. In addition, the three-year relapse-free survival rate was 45.0% in patients with low levels of *CREB3*, while it was 5.0% in patients with high levels of *CREB3* (Figure 2C). The overall survival was also better in the low-*CREB3* group than in the high-*CREB3* group (Figure 2D). These data suggest that *CREB3* is upregulated in glioblastoma and is associated with a poor prognosis.

**Table 1:** CREB3 expression correlate with clinic-pathological parameters of patients with GBM.

**Parameters**	**Number**	**GDF10**	***p* value**
**Age**			0.414
≤ 50	12	0.336 ± 0.220	
> 50	28	0.392 ± 0.201	
**Tumor volume**			
≤ 3 cm	18	0.496 ± 0.251	0.008**
> 3 cm	22	0.226 ± 0.122	
**Ki67**			0.023*
≤ 35%	16	0.447 ± 0.242	
> 35%	24	0.285 ± 0.102	
**Gender**			0.321
Male	11	0.368 ± 0.181	
Female	29	0.315 ± 0.236	
**Distant metastasis**			0.046*
Yes	22	0.421 ± 0.182	
No	18	0.279 ± 0.208	
**WHO stage**			0.006**
I-II	19	0.426 ± 0.311	
III-IV	21	0.261 ± 0.209	

Student’s t test, *P<0.05, **P<0.01.

### The upregulation of CREB3 promoted the proliferation of SHG-44 cells

**Figure 3:**
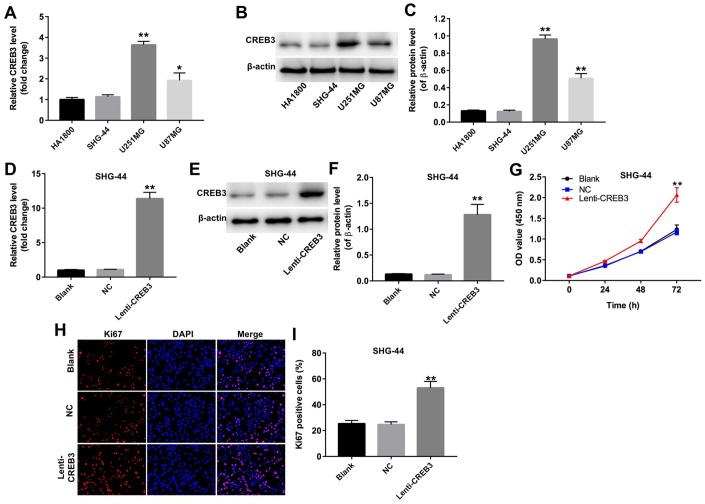
**The upregulation of CREB3 promoted the proliferation of SHG-44 cells.** (**A**) The relative levels of *CREB3* in four cell lines (HA1800, SHG-44, U251MG and U87MG cells) were detected by qRT-PCR. (**B**, **C**) The relative levels of CREB3 in HA1800, SHG-44, U251MG and U87MG cells were detected by Western blotting. β-actin was used as a loading control. (**D**) *CREB3* levels in SHG-44 cells transfected with the NC or lenti-CREB3 were detected by qRT-PCR. (**E**, **F**) CREB3 levels in SHG-44 cells transfected with the NC or lenti-CREB3 were measured by Western blotting. β-actin was used as a loading control. (**G**) The viability of SHG-44 cells transfected with the NC or lenti-CREB3 was detected with a CCK-8 assay at 0, 24, 48 and 72 h. (**H**, **I**) The relative fluorescence levels were quantified for KI67 and DAPI staining. *P<0.05, **P<0.01 compared with the HA1800 group or NC group.

Next, qRT-PCR and Western blotting were used to detect the expression of CREB3 in human astrocytes (HA1800 cells) and three glioblastoma cell lines (SHG-44, U251MG and U87MG). CREB3 was upregulated the most in U251MG cells compared with HA1800 cells; in fact, there was no difference in CREB3 levels between SHG-44 cells and HA1800 cells (Figure 3A–3C).

To determine the function of CREB3 in glioblastoma, we overexpressed CREB3 in SHG-44 cells. CREB3 was markedly upregulated in SHG-44 cells transfected with lenti-CREB3 plasmids (Figure 3D–3F). A CCK-8 assay demonstrated that CREB3 overexpression markedly enhanced cell proliferation compared with NC treatment (Figure 3G). Similarly, an immunofluorescence assay revealed that KI67-positivity was significantly greater among CREB3-overexpressing cells than among NC cells (Figure 3H and 3I). These results indicate that the upregulation of CREB3 promoted the proliferation of SHG-44 cells.

### The downregulation of *CREB3* induced apoptosis in U251MG cells

**Figure 4:**
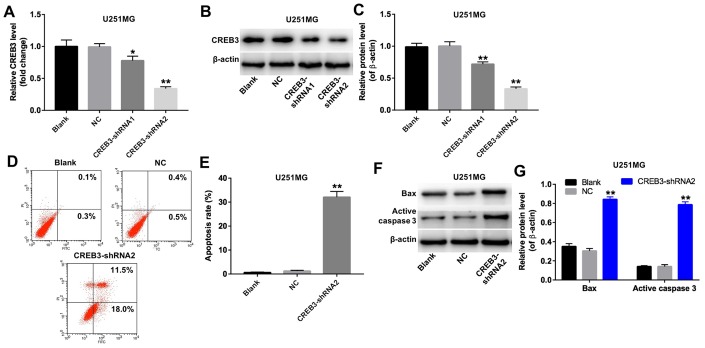
**The downregulation of *CREB3* induced apoptosis in U251MG cells.** (**A**) *CREB3* levels were detected by qRT-PCR in U251MG cells transfected with the NC, CREB3-shRNA1 or CREB3-shRNA1. (**B**, **C**) CREB3 levels were measured by Western blotting in U251MG cells transfected with the NC, CREB3-shRNA1 or CREB3-shRNA2. β-actin was used as a loading control. (**D**, **E**) U251MG cells were transfected with CREB3-shRNA2 for 72 h. Apoptosis was detected with Annexin V and PI double staining, and the percentage of apoptotic cells was calculated. (**F**) BAX and active caspase 3 levels were detected by Western blotting in U251MG cells transfected with CREB3-shRNA2 for 72 h. (**G**) The relative levels of BAX and active caspase 3 were quantified and normalized to β-actin levels. *P<0.05, **P<0.01 compared with the NC group.

We next used two different shRNAs (CREB3-shRNA1 and CREB3-shRNA2) to downregulate *CREB3* in U251MG cells. QRT-PCR and Western blotting illustrated that CREB3-shRNA2 downregulated *CREB3* more significantly than CREB3-shRNA1 in U251MG cells (Figure 4A–4C). The apoptosis rate increased markedly in U251MG cells following their transfection with CREB3-shRNA2 (Figure 4D and 4E). Moreover, the protein levels of BAX and active caspase 3 were greater in U251MG cells transfected with CREB3-shRNA2 than in those transfected with the NC (Figure 4F and 4G). These data illustrate that the downregulation of *CREB3* induced apoptosis in U251MG cells.

### The upregulation of CREB3 increased the invasion of SHG-44 cells

**Figure 5:**
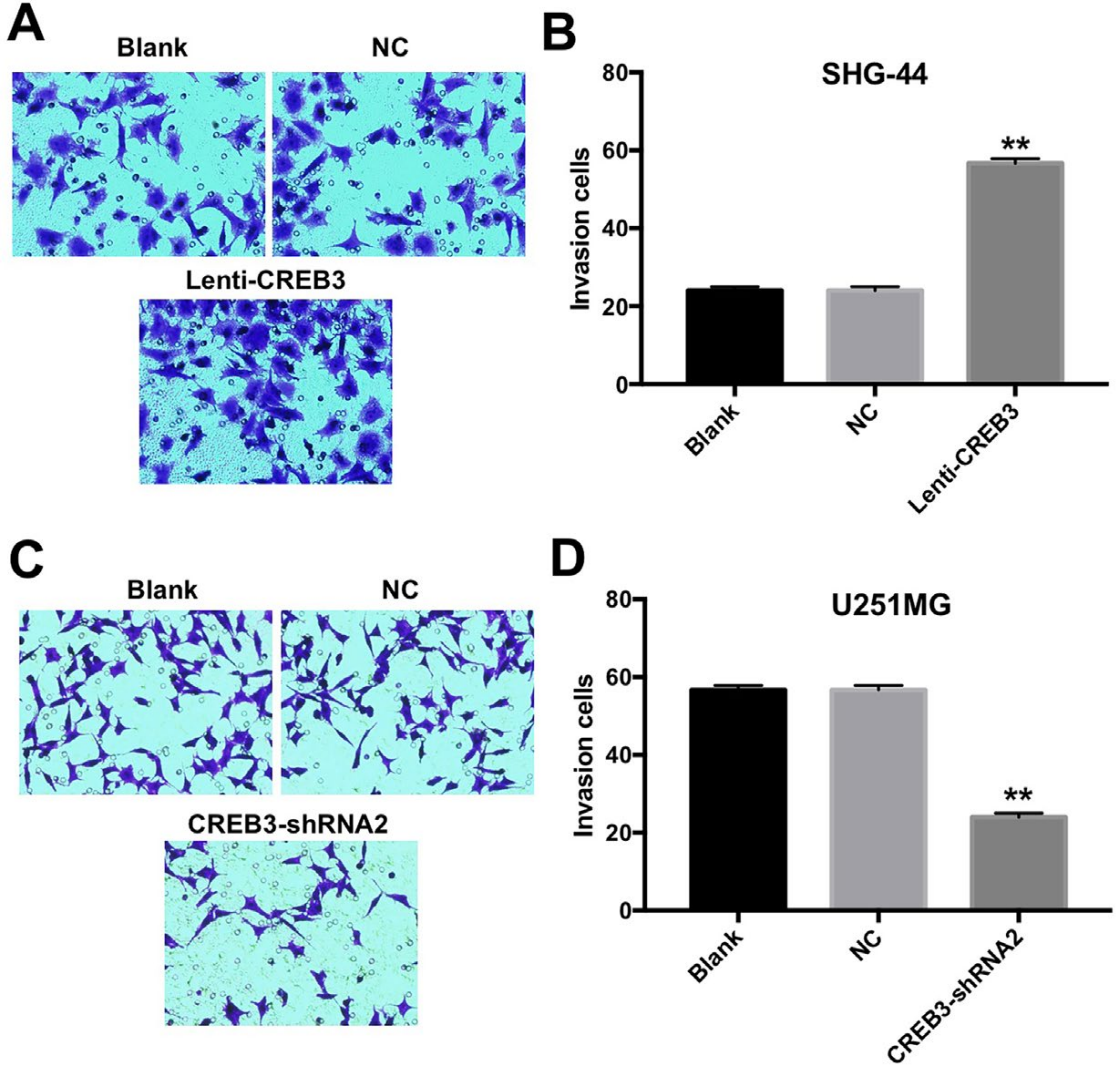
**The upregulation of CREB3 increased the invasion of SHG-44 cells.** (**A**, **B**) The invasive abilities of SHG-44 cells transfected with the NC or lenti-CREB3 were detected with a Transwell assay. (**C**, **D**) The invasive abilities of U251MG cells transfected with the NC or CREB3-shRNA2 were detected with a Transwell assay. **P<0.01 compared with the NC group.

Since glioblastoma is an aggressive type of brain tumor [18], we investigated whether overexpressing or downregulating CREB3 would alter the invasive abilities of glioblastoma cells. As shown in Figure 5A and 5B, the invasive abilities of SHG-44 cells increased significantly after the cells were transfected with lenti-CREB3 plasmids. On the other hand, the downregulation of *CREB3* markedly inhibited the invasion of U251MG cells compared with NC treatment (Figure 5C and 5D).

### The downregulation of CREB3 activated ERS pathway proteins in U251MG cells

**Figure 6:**
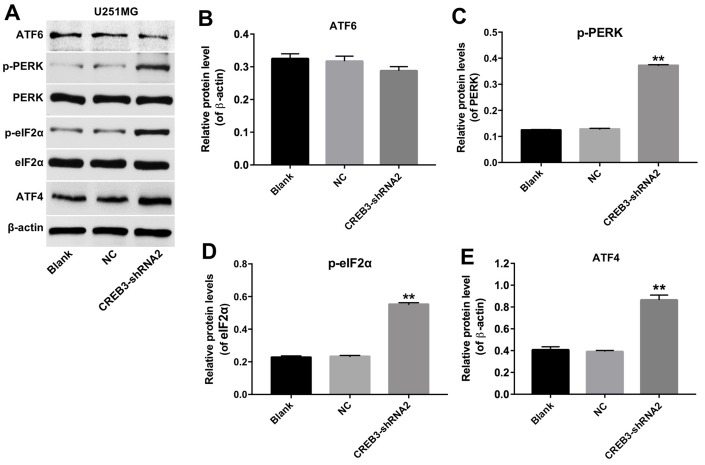
**The downregulation of *CREB3* activated ERS pathway proteins in U251MG cells.** (**A**) The levels of the ERS-related proteins ATF6, p-PERK, p-eIF2α and ATF4 were detected in U251MG cells transfected with the NC or CREB3-shRNA2. (**B**–**E**) The relative levels of ATF6 (**B**), p-PERK (**C**), p-eIF2α (**D**) and ATF4 (**E**) were quantified and normalized to β-actin, PERK, eIF2α and β-actin levels respectively. **P<0.01 compared with the NC group.

**Figure 7:**
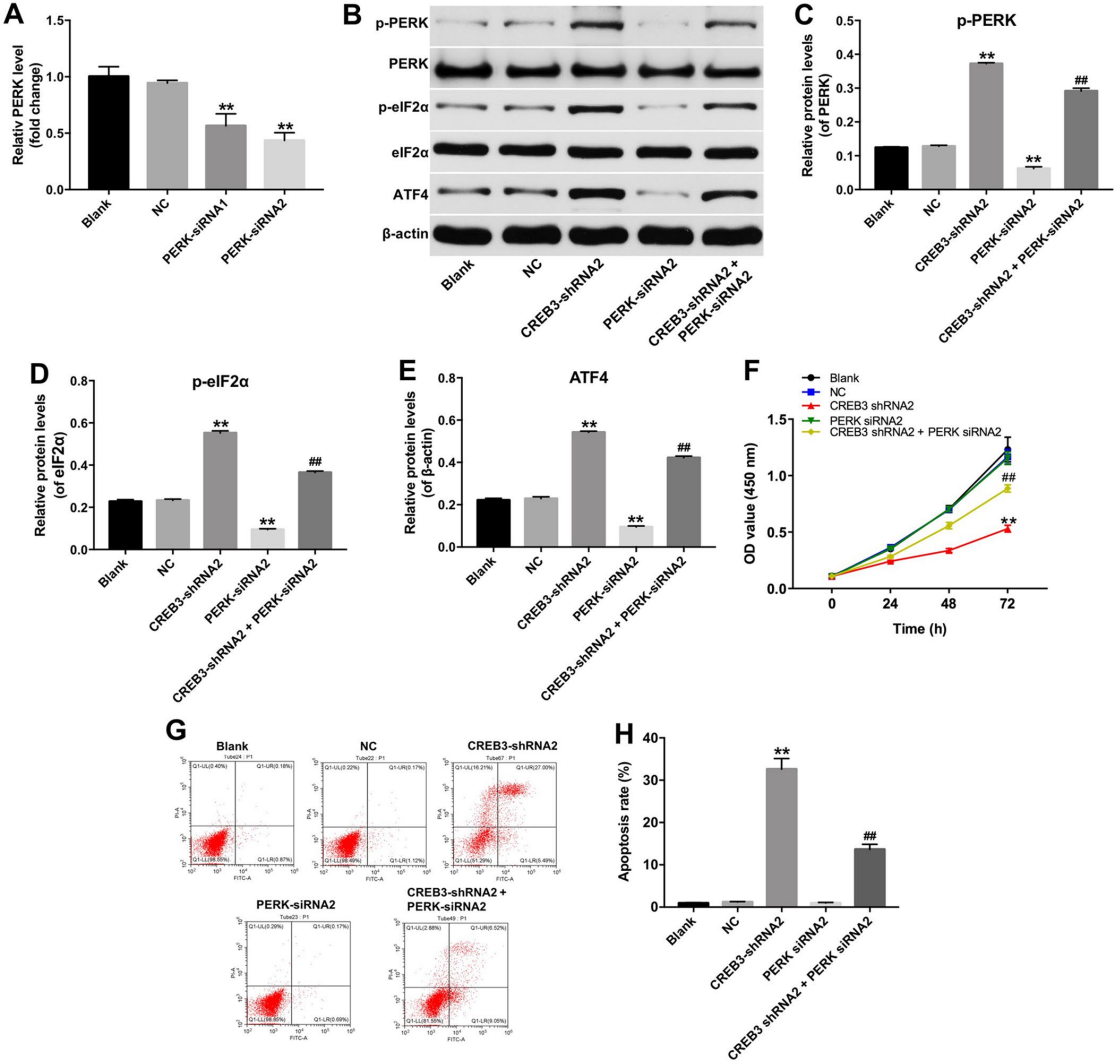
**The downregulation of *CREB3* inhibited growth of U251MG cells via activating ERS pathway.** (**A**) PERK levels were detected by qRT-PCR in U251MG cells transfected with the NC, PERK-siRNA1 or PERK-siRNA2. (**B**) The levels of the ERS-related proteins p-PERK, p-eIF2α and ATF4 were detected in U251MG cells transfected with the CREB3-shRNA2, PERK-siRNA2 or co-transfected with CREB3-shRNA2 and PERK-siRNA2. (**C**–**E**) The relative levels of p-PERK (**C**), p-eIF2α (**D**) and ATF4 (**E**) were quantified and normalized to PERK, eIF2α and β-actin levels respectively. (**F**) The viability of U251MG cells was detected with a CCK-8 assay. (**G**, **H**) Apoptosis was detected with Annexin V and PI double staining, and the percentage of apoptotic cells was calculated. **P<0.01 compared with the NC group, ^##^P<0.01 compared with the CREB3-shRNA2 group.

We next explored whether the inhibition of *CREB3* induced apoptosis in glioblastoma cells by regulating the ERS pathway. As indicated in Figure 6A–6E, the downregulation of *CREB3* significantly increased the protein levels of p-PERK, p-eIF2α and ATF4 compared with NC treatment. In addition, as shown in Figure 7A, the level of PERK was markedly decreased following transfection with PERK-siRNA2. Moreover, downregulation of CREB3 significantly increased the protein levels of p-PERK, p-eIF2α and ATF4 in U251MG cells, which were obviously reversed following transfection with PERK-siRNA2 (Figure 7B–7E). Furthermore, downregulation of CREB3 obviously inhibited the proliferation of U251MG cells, while downregulated PERK in CREB3-shRNA2 treated cells, significantly reversed the growth-inhibitory role of CREB3-shRNA2 in U251MG cells (Figure 7F). Meanwhile, downregulation of CREB3 obviously induced apoptosis of U251MG cells, which was markedly reversed following transfection with PERK-siRNA2 (Figure 7G and 7H). These results suggest that the downregulation of *CREB3* induced apoptosis in U251MG cells by activating ERS pathway proteins.

### The downregulation of *CREB3* inhibited the tumor growth of U251MG xenografts *in vivo*

**Figure 8:**
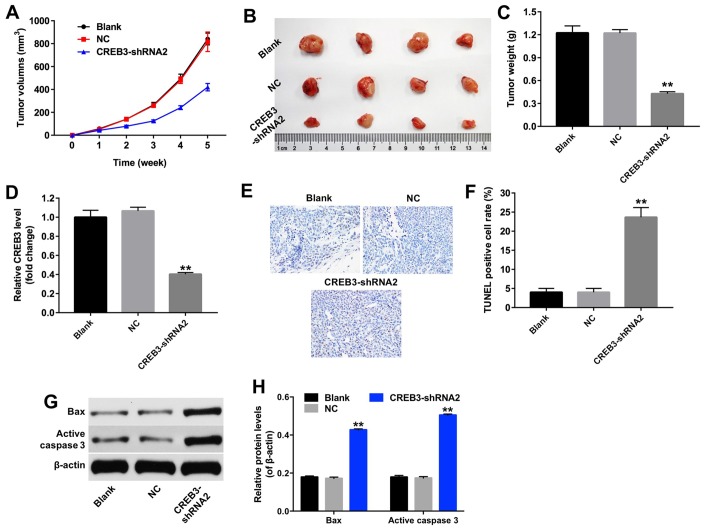
**The downregulation of *CREB3* inhibited U251MG xenograft tumor growth *in vivo*.** (**A**) Mice were subcutaneously implanted with CREB3-shRNA2-infected U251MG cells, and the xenograft tumor volumes were measured weekly. (**B**) Tumors were excised from the xenografts and photographed after five weeks. (**C**) The weights of the tumors were calculated. (**D**) The *CREB3* levels in tumor tissues were detected by qRT-PCR. (**E**, **F**) TUNEL staining was performed on tumor tissues from each group, and the percentage of TUNEL-positive cells was calculated. (**G**) The levels of the Bax and active caspase 3 in tumor tissues were detected by Western blotting. (**H**) The relative levels of Bax and active caspase 3 were quantified and normalized to β-actin levels. **P<0.01 compared with the NC group.

**Figure 9:**
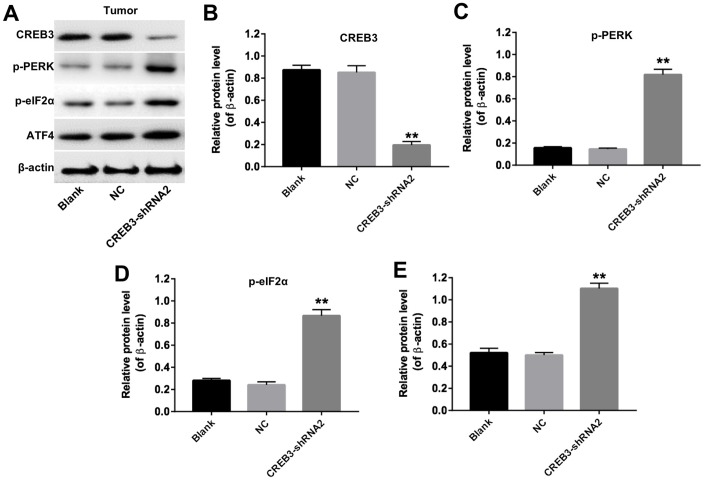
**The downregulation of *CREB3* activated ERS pathway proteins in tumor tissues.** (**A**) The levels of *CREB3*, p-PERK, p-eIF2α and ATF4 in tumor tissues were detected by Western blotting. (**B**–**E**) The relative levels of *CREB3* (**B**), p-PERK (**C**), p-eIF2α (**D**) and ATF4 (**E**) were quantified and normalized to β-actin levels. **P<0.01 compared with the NC group.

We then established a xenograft model to further assess the effects of CREB3 on glioblastoma tumor growth *in vivo*. As indicated in Figure 8A and 8B, the downregulation of *CREB3* significantly repressed xenograft tumor growth. The tumor weights were markedly lower in the CREB3-shRNA2 group than in the NC group (Figure 8C). The relative mRNA levels of *CREB3* were notably lower in CREB3-shRNA2-transfected tumor tissues than in NC-transfected tissues (Figure 8D). Additionally, a TUNEL assay demonstrated that apoptosis was induced in the CREB3-shRNA2 group compared with the NC group (Figure 8E and 8F). Meanwhile, we tested the expressions of Bax and active caspase 3 in tumor tissues *in vivo*. The data indicated that knockdown of CREB3 significantly upregulated the protein levels of Bax and active caspase 3 in tumor tissues (Figure 8G and 8H). As shown in Figure 9A–9E, the downregulation of *CREB3* significantly increased the protein levels of p-PERK, p-eIF2α and ATF4 *in vivo*. These data suggest that the downregulation of *CREB3* inhibited U251MG xenograft tumor growth *in vivo* by activating ERS pathway proteins.

## DISCUSSION

Relatively few biomarkers have been applied to cancer diagnosis, therapy and prognostic evaluation, so it is important to explore new biomarkers to enhance the treatment and prognostic assessment of cancer patients [19]. In this study, GO and KEGG analyses were used to identify DEGs in patients with glioblastoma. One of these DEGs was *CREB3*, which is known to promote the malignant progression of glioblastoma [2].

CREB3 is most highly expressed in the hypothalamus, hippocampus and anterior pituitary [20]. Xue et al. found that the downregulation of *CREB3* inhibited the growth of glioblastoma [2]. In the present study, *CREB3* mRNA levels were found to be significantly greater in glioblastoma tumor tissues than in adjacent normal tissues. Additionally, lower levels of *CREB3* were associated with higher relapse-free and overall survival rates in patients with glioblastoma. Thus, CREB3 may function as a tumor promoter in glioblastoma patients.

To assess the effects of CREB3 in glioblastoma, we employed a human astrocyte cell line (HA1800) and three glioblastoma cell lines (SHG-44, U251MG and U87MG). Our qRT-PCR and Western blot data indicated that CREB3 levels were greater in U251MG cells than in HA1800 cells, but were similar in SHG-44 and HA1800 cells. Therefore, we transfected SHG-44 cells with a CREB3 overexpression lentivirus, and transfected U251MG cells with CREB3-shRNA2. The overexpression of CREB3 promoted the proliferation and invasion of SHG-44 cells, while the downregulation of *CREB3* inhibited the tumor growth and invasion of U251MG cells. In addition, the silencing of *CREB3* induced apoptosis in U251MG cells and increased the levels of BAX and active caspase 3. A previous report indicated that the knockdown of CREB3 regulatory factor (*CREBRF*) inhibited cell proliferation by suppressing AKT signaling [21]. This evidence supports the notion that CREB3 functions as a tumor promoter.

CREB3 is an ERS-related protein [22, 23] that downregulates the UPR as a membrane-bound basic leucine zipper transcription factor [24]. The UPR is primarily regulated by three ER-membrane-bound proteins – PERK, IRE1 and ATF6 – the latter of which also contains a basic leucine zipper domain [24]. We found that knocking down *CREB3* increased p-PERK, p-eIF2α and ATF4 levels and induced apoptosis *in vitro* and *in vivo*. A previous study suggested that the inhibition of IL-6 could induce apoptosis by regulating the CREBRF-CREB3-ATG5 pathway [25]. The upregulation of CREB3 has been reported to protect cells against ERS-induced apoptosis [23]. Ma et al. indicated that the activation of PERK-eIF2α-ATF4 could induce apoptosis in glioma cells [26]. Therefore, the knockdown of *CREB3* may have induced apoptosis in U251MG cells *in vitro* and *in vivo* by activating ERS pathway proteins.

In conclusion, we demonstrated that *CREB3* was upregulated in patients with glioblastoma. In glioblastoma cells, the overexpression of CREB3 increased proliferation and invasion, while the downregulation of *CREB3* induced apoptosis and inhibited invasion. In addition, the knockdown of *CREB3* activated ERS pathway proteins and induced apoptosis in U251MG cells *in vitro* and *in vivo*. These data suggest a novel mechanism whereby CREB3 may function as a tumor promoter by suppressing the ERS pathway in patients with glioblastoma. However, the complete mechanism by which knocking down *CREB3* induces apoptosis in glioblastoma requires further investigation.

## MATERIALS AND METHODS

### Clinical samples

Sixty pairs of glioblastoma tissue samples and adjacent normal tissues were obtained from the Affiliated Hospital of Guizhou Medical University. Patients were recruited from May 5, 2017 until July 25, 2018. Patients met the inclusion criteria if they were 18 to 70 years old and had been diagnosed with glioblastoma of World Health Organization criteria grade I to IV. The present study was approved by the Ethics Committee of the Affiliated Hospital of Guizhou Medical University.

### RNA sequencing

For RNA sequencing, total RNA was extracted from glioblastoma tissues and adjacent normal tissues with Trizol (Invitrogen, Carlsbad, CA, USA) according to the manufacturer’s instructions. A Nanodrop 2000 spectrophotometer (Thermo Fisher Scientific) was used to determine the purity and quality of the total RNA samples. Small RNA libraries were generated with a Small RNA Library Preparation Kit (Illumina, CA, USA) [27]. quantitative real-time PCR (qRT-PCR) were used to analyze the RNA sequences.

### GO and KEGG pathway analyses

GO (http://www.geneontology.org/) and KEGG (http://www.genome.jp/kegg/) enrichment analyses are widely used to determine potential gene annotations. These online bioinformatics tools allow the functional analysis of extensive lists of genes and proteins. In the present study, DEGs (http://mathworld.wolfram.com) were obtained by hypergeometric tests. GO analysis was used to allocate protein biomarkers into their corresponding pathways at three levels: biological process, cellular component and molecular function. The criteria used for GO and KEGG analyses were P- values *≤* 0.05. Then, a volcano map was generated.

### Cell culture

The human astrocyte cell line HA1800 and glioblastoma cell lines SHG-44, U251MG and U87MG were purchased from the American Type Culture Collection (ATCC, Rockville, MD, USA). All these cell lines were cultured in Roswell Park Memorial Institute (RPMI)-1640 medium (Gibco, USA) supplemented with 10% fetal bovine serum (Gibco), 100 U/mL penicillin and 100 U/mL streptomycin (Thermo Fisher Scientific) in a humidified incubator with 5% CO_2_ at 37°C.

### qRT-PCR

Trizol reagent (Invitrogen) was used to extract total RNA from cells in accordance with the manufacturer’s procedure. Next, a cDNA synthesis kit (Invitrogen) was used to synthesize cDNA. Then, qRT-PCR was performed with a SYBR Premix Ex Taq II kit (TaKaRa, Dalian, China) on an ABI 7900HT instrument (ABI, NY, USA). The qRT-PCR reactions were performed at 95°C for 5 min, followed by 35 cycles of 95°C for 30 s, 60°C for 45 s and 72°C for 30 s. The following primers were used: *CREB3*: F: 5′-CTTCTCCGACTCCAACCTTC-3′, R: 5′-CCACATCCTCACACCTAACC-3′; *GAPDH*: F: 5′-CG GACCAATACGACCAAATCCG-3′, R: 5′-AGCCACA TCGCTCAGACACC-3′. The primers were purchased from GenePharma (Shanghai, China). The relative expression of *CREB3* was calculated by the 2^-ΔΔCT^ method.

### Lentiviral plasmids construction

Negative control (NC, scrambled shRNA), CREB3-shRNA1 (UUUCUGAGCAGUGUAUCAUAUUGGG) and CREB3-shRNA2 (CAGGAGATGTCTAGGCT GAT) lentiviruses were purchased from GenePharma (Shanghai, China). Meanwhile, the CREB3 sequence was synthesized by GenePharma (Shanghai), and then sub-cloned into the pWPXL (lentiviral expression vector). Then, 293T cells were transduced with the NC, pWPXL-CREB3 (Lenti-CREB3) or CREB3-shRNA1/2 plasmids for 48 h. Forty-eight hours later, the supernatants were collected and filtered with a low-protein-binding syringe filter.

### U251MG shRNA knockdown

U251MG cells (4x10^5^ cells per well) were seeded into 60-mm cell plates overnight. Then, the CREB3-shRNA supernatants were added to the cells. Twenty-four hours later, the virus-containing medium was replaced with fresh complete medium. Puromycin (2.5 μg/mL, Sigma Aldrich, St. Louis, MO, USA) was used for three days to select stable U251MG cells. Then, qRT-PCR and Western blotting assays were used to determine the expression of CREB3 in the cells.

### Exogenous CREB3 overexpression

SHG-44 cells (4x10^5^ cells per well) were seeded into 60-mm cell plates overnight. Then, lenti-CREB3 supernatants were added to the SHG-44 cells for 24 h. Twenty-four hours later, the virus-containing medium was replaced with fresh complete medium. Puromycin (2.5 μg/mL, Sigma Aldrich) was used for three days to select stable SHG-44 cells. Then, qRT-PCR and Western blotting assays were used to determine the expression of CREB3 in the cells.

### Cell transfection

PERK-siRNA1, PREK-siRNA2 and NC were purchased from GenePharma (Shanghai, China). U251MG cells were transfected with PERK-siRNA1, PREK-siRNA2 and NC for 72 h, using lipofectamine 2000 transfection reagent (Invitrogen, Carlsbad, CA, USA) following the manufacture’s instruction.

### Western blot analysis

Protein was extracted from SHG-44 and U251MG cells, and a bicinchoninic acid assay (Thermo Fisher Scientific) was used to determine the protein concentration. Proteins (40 μg) were electrophoretically separated on 10% sodium dodecyl sulfate polyacrylamide gels, and then were transferred onto polyvinylidene fluoride membranes (Thermo Fisher Scientific). After being blocked with 5% non-fat milk with Tris-buffered saline Tween at 37°C, the membranes were incubated with the following primary antibodies overnight at 4°C: anti-CREB3 (1:1000), anti-BAX (1:1000), anti-active caspase 3 (1:1000), anti-ATF6 (1:1000), anti-p-PERK (1:1000), anti-PERK (1:1000), anti-p-eIF2α (1:1000), anti-eIF2α (1:1000), anti-ATF4 (1:1000) and anti-β-actin (1:1000). Subsequently, the blots were incubated with an anti-rabbit secondary antibody (1:5000) at 37°C for 2 h. Protein levels were quantified with ImageJ 1.50i software (National Institutes of Health, Bethesda, MD, USA).

### Cell counting kit 8 (CCK-8) assay

SHG-44 cells were plated at 2x10^4^ cells per well in a 96-well plate for 24 h before the assay. Cells were exposed to lenti-CREB3 for 24, 48 or 72 h. Then, 10 μL of the CCK-8 reagent (Beyotime, Shanghai, China) was added to each well, and the cells were incubated for 2 h at 37°C. Finally, the absorbances were measured at 450 nm on a microplate reader (Awareness Technology ChroMate® Microplate Reader).

### Immunofluorescence

SHG-44 cells were plated into 24-well plates overnight. Then, the cells were transfected with the NC or lenti-CREB3 plasmids and incubated for 72 h. Subsequently, the cells were washed twice in phosphate-buffered saline and fixed with 4% polyoxymethylene for 10 min. The cells were incubated with an anti-KI67 primary antibody (Abcam; ab15580) (1:1000) and stained with 4’,6-diamidino-2-phenylindole (DAPI, ab104139) at 4°C overnight. Then, the cells were incubated with a secondary antibody at 37°C for 1 h, and were observed under a microscope.

### Flow cytometric analysis of cell apoptosis

U251MG cells were resuspended in a binding buffer at a concentration of 5x10^5^ cells/mL. Then, the cells were treated with 10 μL of Annexin V-fluorescein isothiocyanate (FITC) and propidium iodide (PI). The cells were vortexed and incubated for 15 min at room temperature. Apoptotic cells were quantified with a BD Accuri™ C6 flow cytometer (BD Biosciences). BD Accuri™ C6 software (BD Biosciences) was used to analyze the results.

### Matrigel invasion assay

Cell invasion was assessed in Transwell chambers (24-well type, 8 μm pore size; Corning Incorporated, Corning, NY, USA). Briefly, the upper chamber was pre-treated with 100 μL of Matrigel. Then, SHG-44 or U251MG cells were seeded into the upper chamber with serum-free medium, while RPMI-1640 medium containing 10% fetal bovine serum was added to the lower chamber. The plates were incubated for 24 h at 37°C to allow the cells on the upper surface of the filter to migrate to the lower chamber. Then, the cells that had migrated were fixed in 100% methanol and stained with 0.05% crystal violet at room temperature. The invading cells were visualized and counted in three randomly selected fields.

### Animal study

Five-week-old male nude mice (n=24) were obtained from the Laboratory Animal Center of China (Shanghai, China). U251MG cells (5x10^6^ cells in 100 μL of phosphate-buffered saline) with one of three different treatments (blank, NC or CREB3-shRNA2) were subcutaneously injected into the right armpit area of each mouse. The tumor volume was measured with Vernier calipers every week according to the formula: volume = (length × (width^2^)) / 2. After five weeks, the mice were sacrificed. The entire tumor from each mouse was excised, weighed and used for TUNEL staining experiments. All animal experiments were performed in accordance with our institutional guidelines, following a protocol approved by the Ethics Committees of the Affiliated Hospital of Guizhou Medical University.

### TUNEL staining

Tumor tissue sections were stained with an APO-BrdU™ TUNEL Assay Kit (A23210, Thermo Fisher Scientific) in accordance with the manufacturer’s instructions.

### Statistical analysis

For each analysis, at least three independent experiments were performed, and all data were expressed as the mean ± standard deviation (SD). Statistical analyses were conducted with SPSS v21.0 (IBM Corp., Armonk, NY, USA) and GraphPad Prism v5.0 (GraphPad Software, Inc., La Jolla, CA, USA). Comparisons between two groups were performed with Student’s t-test, while comparisons among multiple groups were performed with one-way analysis of variance followed by Dunnett’s test. P<0.05 or P<0.01 was considered to indicate a statistically significant difference.
